# Erlotinib sensitivity of *MAPK1*p.D321N mutation in head and neck squamous cell carcinoma

**DOI:** 10.1038/s41525-020-0124-5

**Published:** 2020-04-20

**Authors:** Hoi-Lam Ngan, Peony Hiu Yan Poon, Yu-Xiong Su, Jason Ying Kuen Chan, Kwok-Wai Lo, Chun Kit Yeung, Yuchen Liu, Eileen Wong, Hui Li, Chin Wang Lau, Wenying Piao, Vivian Wai Yan Lui

**Affiliations:** 1 School of Biomedical Sciences, Faculty of Medicine, The Chinese University of Hong Kong, Hong Kong SAR, Hong Kong; 2 Department of Oral and Maxillofacial Surgery, Faculty of Dentistry, The University of Hong Kong, Hong Kong SAR, Hong Kong; 3 Department of Otorhinolaryngology, Head & Neck Surgery, Faculty of Medicine, The Chinese University of Hong Kong, Hong Kong SAR, Hong Kong; 4 Department of Anatomical and Cellular Pathology, Faculty of Medicine, The Chinese University of Hong Kong, Hong Kong SAR, Hong Kong; 5 Department of Otorhinolaryngology Head and Neck, Yan Chai Hospital, Hong Kong SAR, Hong Kong

**Keywords:** Cancer genetics, Targeted therapies, Head and neck cancer

## Abstract

Head and neck squamous cell carcinoma (HNSCC) lacks predictive biomarkers for drug responses. By targeted sequencing, we identified two *MAPK1* mutations in recurrent HNSCC, *MAPK1*p.D321N, and p.R135K. We previously reported an exceptional erlotinib responder with *MAPK1*p.E322K. Here, by in silico and drug studies, we determined functions of these two recurrence-associated *MAPK1* mutations. Residues D321, R135, and E322 are in 3D proximity. *MAPK1*p.D321N drives marked in vivo erlotinib sensitivity, while p.R135K’s effect is moderate.

## Introduction

Erlotinib is an FDA-approved agent for the treatment of epidermal growth factor receptor (*EGFR*)-mutated non-small cell lung cancer (NSCLC), and pancreatic cancer. Particularly for *EGFR*-mutated NSCLC, remarkable increase in progression-free survival was observed in phase III trials^1,2^. In polycythemia vera, some *JAK2*p.V617-mutated patients have demonstrated erlotinib sensitivity^3^. Recently, early clinical trial data in head and neck squamous cell carcinoma (HNSCC) revealed the presence of an *EGFR-AS1*(c.2361G>A) synonymous mutation^4^, high baseline phospho-MAPK (ref. ^5^) and *MAPK1*p.E322K mutation^6^ as additional potential biomarkers for erlotinib sensitivity. As HNSCC lacks predictive biomarkers for drug responses, in-depth studies were conducted on *MAPK1*p.E322K, the mutation found in a complete erlotinib responder. Subsequent results revealed *MAPK1*p.E322K’s ability to hyperactivate EGFR, which could confer erlotinib sensitivity in HNSCC (refs ^6,7^).

HNSCC frequently recurs. Once recurred, patients have dismal survivals of only ~7 months^8^. Here, by targeted sequencing, we have identified two *MAPK1* somatic mutations, p.D321N and p.R135K, in two cases of primary–recurrent HNSCC from Hong Kong. We aimed to determine if these two recurrence-associated *MAPK1* mutations may also confer erlotinib sensitivity in HNSCC as reported for *MAPK1*p.E322K (refs ^6,7^). Our findings show that *MAPK1*p.D321N confers heightened sensitivity to erlotinib in vivo, while p.R135K’s effect is moderate.

## Results

### Potential high rate of *MAPK1* mutations in Asian HNSCC

**Fig. 1:**
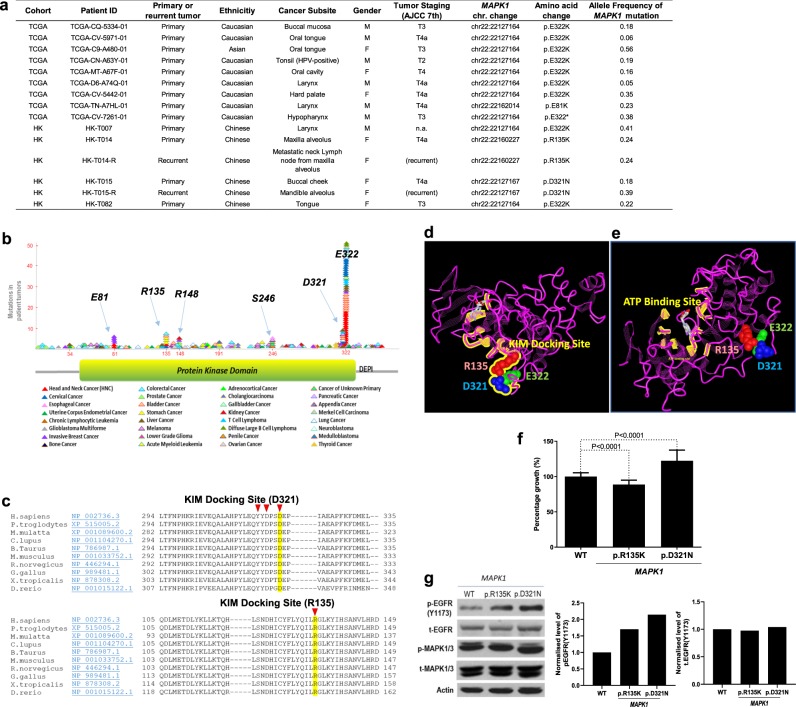
*MAPK1* mutations found in Asian HNSCC are drivers for growth. **a** Table showing HNSCC cases with somatic *MAPK1* mutations in the US-TCGA-HNSCC Provisional cohort (*N* = 512 tumors) and the Asian HK-HNSCC cohort (*N* = 105 tumors). **b** Mapping of mutation sites of the *MAPK1* gene based on the pan-cancer data from TCGA (refs ^9,10^) and the COSMIC database (ref. ^11^). Each mutational event is represented by one triangular symbol. Color annotation of various cancer types are shown at the bottom. **c** Conserved regions of the MAPK1 (ERK2) proteins across species around amino acid positions p.D321 and p.R135 are shown. The amino acid residues of the KIM-docking site are indicated by red arrows. **d** The X-ray crystallography structure of the human MAPK1 (ERK2) protein (locked with the ATP competitive inhibitor 5-Iodotubercidin and the allosteric inhibitor peptide-type ERK2 inhibitor; PDB ID: 5AX3 (ref. ^13^); MMDB ID: 136379 (ref. ^14^). Amino acid residues R135, D321, and E322 are highlighted in red, blue, and green, respectively. Residue R135 is 9.0 Å away from E322 and 11.3 Å away from D321. The peptide sequence of the KIM domain is highlighted and labeled in yellow. **e** The same X-ray crystallography structure of MAPK1 protein showing the peptide sequence of the ATP-binding domain highlighted in yellow, and the ATP molecule shown in gray color. **f** Driver activity assay, by MTT assay, of FaDu cells that ectopically expressed *MAPK1*-WT, *MAPK1*p.D321N, and *MAPK1*p.R135K mutants. Cells were seeded on a 48-well plate at a density of 1.2 × 10^4^ cell/well with DMEM and 5% FBS. MTT assay were conducted at 96 h after seeding. A cumulative graph of three independent repeats is shown (total *N* ≥ 14 wells). Driver activity was normalized against *MAPK1*-WT. The *MAPK1*p.D321N is a driver for FaDu cell growth (*P* < 0.0001; 88.65% ± 1.262 SEM), while the *MAPK1*p.R135K moderately suppresses cell growth (*P* < 0.0001; 122.3% ± 4.060 SEM). **g** Western blotting showing the level of p-EGFR(Y1173), t-EGFR, p-MAPK, and t-MAPK in FaDu cells expressing *MAPK1*-WT, *MAPK1*p.R135K, and *MAPK1*p.D321N mutants, respectively. The p-EGFR and total EGFR levels were normalized to actin, and shown as bar graphs. Three independent repeats were performed and all repeats showed similar trends.

In 32 The Cancer Genome Atlas (TCGA) pan-cancers^9,10^, the average *MAPK1* mutation rate is 0.79% (86/10,953 cases, 32 TCGA pan-cancers, as of August 2019). Notably, the *MAPK1* mutation rate in HNSCC appears to be relatively higher (1.8%; 9/512 cases) than that in the TCGA pan-cancers, and such HNSCC-associated *MAPK1* mutations are almost all uniformly p.E322K or p.E322* mutations (Fig. 1a). Interestingly, a relatively diverse *MAPK1* mutation pattern and a relatively higher mutation rate of *MAPK1* (5.7%; 6/105 fresh frozen tumors from 103 unique individuals) were identified in our small Hong Kong HNSCC cohort (by targeted sequencing, >500 × mean depth covering 92.2% of all nine *MAPK1* exons). No germline mutations are found. Importantly, among which, two patients bore primary-to-recurrence somatic *MAPK1* mutations, namely *MAPK1*p.D321N and p.R135K mutations (Fig. 1a). For the HK-T015 patient, his recurrent tumor carried an apparent increase of *MAPK1*p.D321N allele frequency from 18.27% (primary) to 39.14% (recurrent), suggestive of a likely driver activity during recurrence. Whereas the allele frequency of *MAPK1*p.R135K from primary to recurrent tumor did not change in the other patient, HK-T014.

### Residues E322, D321N, and R135K in 3D proximity

We mapped all *MAPK1* somatic mutations from pan-cancers^9–11^, and identified *MAPK1* hotspot mutation cluster regions (arbitrarily defined in this study as mutation sites with >5 mutations) at amino acid residues E322 and D321, followed by the lesser frequent mutation cluster regions at E81, R135, R148, and S246 of the MAPK1 (ERK2) protein (Fig. 1b). D321 resides on the same DEP-conserved sequence as E322, which is located right near the highly conserved kinase interaction motif (KIM) of *MAPK1* across species (Fig. 1c). KIM-docking domain is a conserved functional domain among all MAPKs known to be involved in kinase interactions^12^. To further understand the potential impact of HNSCC-associated *MAPK1* hotspot mutations (p.E322K, p.D321N, and p.R135K) in relation to the ERK2 protein structure, we mapped the 3D locations of residues E322, D321, and R135 on the resolved X-ray crystallography structure of the human MAPK1 (ERK2) (the structure was resolved with an ATP competitive inhibitor 5-Iodotubercidin and the allosteric inhibitor peptide-type ERK2 inhibitor; PDB ID: 5AX3 (ref. ^13^); MMDB ID: 136379 (ref. ^14^)). Strikingly, all three residues cluster in close 3D proximity of only 9.0–12.8 Å from each other (but distant from the ATP-binding site), and all are located on the “exposed” surface of ERK2 and belong to the KIM-docking domain of MAPK1, indicating that mutations of these residues potentially affect MAPK1’s protein interactions with other kinases (Fig. 1d, e).

### *MAPK1*p.D321N and p.R135K drive EGFR activation

Next, we examined the potential driver activity of these two mutations in vitro. Ectopic expression of *MAPK1*p.D321N and p.R135K in FaDu cells revealed opposite driver activities of these mutations. *MAPK1*p.D321N was a driver for HNSCC cell growth (22.3% growth increase vs. *MAPK1*-wild type (WT); *P* < 0.0001), while *MAPK1*p.R135K was unexpectedly a moderate suppressor for HNSCC cell growth (11.4% growth inhibition vs. *MAPK1*-WT, *P* < 0.0001; Fig. 1f). Besides differences in their driver activities, these two mutations also demonstrated differential ability to activate EGFR. Ectopic expressions of *MAPK1*p.D321N in FaDu resulted in 2.14-fold increase in EGFR(Y1173) phosphorylation, a well-known EGFR transphosphorylation site for its activation, while a relatively lower level of EGFR activation was noted with *MAPK1*p.R135K (Fig. 1g, original uncropped images of the blots were shown in Supplementary Fig. 1). Our finding that *MAPK1*p.D321N is a potent driver with high level of EGFR activation suggests functionally similarities between p.D321N and our previously reported p.E322K mutation that caused heightened sensitivity to erlotinib in the HNSCC exceptional responder^6^.

### *MAPK1*p.D321N is erlotinib-sensitive in vivo

**Fig. 2:**
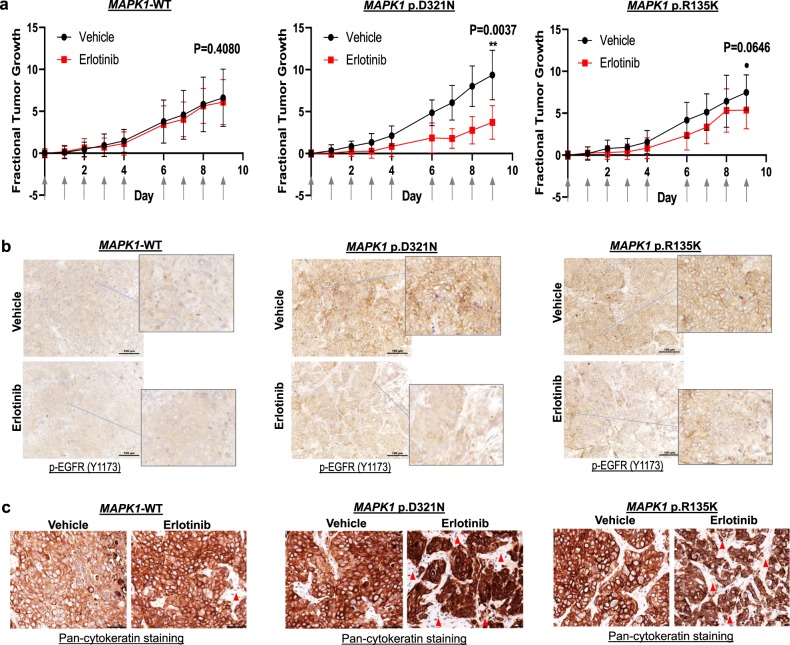
*MAPK1*p.D321N mutation confers erlotinib sensitivity in vivo. **a** Fractional tumor growth curves of in vivo tumor expressing *MAPK1-*WT, *MAPK1*p.D321N, or *MAPK1*p.R135K (mean tumor sizes with SEM). FaDu cells expressing *MAPK1*-WT, *MAPK1*p.D321N, and *MAPK1*p.R135K, respectively, were inoculated into nude mice subcutaneously (8 × 10^5^ cells per inoculation). Mice with tumor expressing respective *MAPK1*-WT/mutations were randomized into erlotinib (erlotinib dissolved in 10% HP-β-CD) or vehicle (10% HP-β-CD) treatment groups (*N* = 8 tumors per group). Treatment started when tumors were palpable and reached the size ~3 × 3 – 4 × 4 mm^2^. Erlotinib or the vehicle control were administered by oral gavage (50 mg/kg erlotinib or the corresponding vehicle amount) for five consecutive days as indicated by the arrows on the *X*-axis. **b** IHC staining showing membranous p-EGFR expression of these xenografts post erlotinib/vehicle treatments (*N* > 3 per group). A total of 100 µm scale bars were shown. **c** IHC staining showing corresponding pan-cytokeratin expression in these tumors post erlotinib/vehicle treatment (*N* > 3 per group). A total of 50 µm scale bars were shown.

Prompted by our previous finding that *MAPK1*p.E322K could demonstrate heightened sensitivity to erlotinib in vivo reminiscent of the patient’s clinical response in the exceptional responder, we generated isogenic tumor xenografts expressing MAPK1-WT, *MAPK1*p.D321N, and *MAPK1*p.R135K, and compared their in vivo erlotinib responses. As shown by Fig. 2b, xenografts of the *MAPK1*p.D321N and p.R135K mutants both showed 80–90% cells with membranous staining of p-EGFR demonstrative of activated EGFR, while only 1% positivity was noted in the *MAPK1*-WT xenografts, which was consistent with our in vitro findings that both mutants were capable of activating EGFR (Fig. 1g). For mice bearing FaDu-*MAPK1-*WT xenografts, erlotinib treatment did not result in any change in tumor size or membranous p-EGFR signal in the tumors (vs. vehicle control, *P* = 0.4080; Fig. 2a, b). Importantly, for *MAPK1*p.D321N mutant tumors, erlotinib treatment resulted in a significant reduction in tumor volume (60.3% reduction vs. vehicle treatment, *P* = 0.0037; Fig. 2a), with concomitant increases in tumor-negative areas (i.e., cytokeratin-negative areas; Fig. 2c), as well as dramatic decreases in activated p-EGFR (membranous) in the tumors with <20% of membranous p-EGFR signals remaining (Fig. 2b). Of note, among *MAPK1*p.R135K mutant xenografts, erlotinib treatment caused a trend for tumor size reduction (28.0% reduction vs. vehicle treatment; *P* = 0.0646; Fig. 2a), with noticeable increases in tumor-negative areas (Fig. 2c, cytokeratin-negative area). Notably, a moderate reduction of percent tumor cells bearing membranous p-EGFR staining was observed (50% vs. 90% in the vehicle-treated *MAPK1*p.R135K mutant tumors, Fig. 2b). Thus, we demonstrated a heightened sensitivity of *MAPK1*p.D321N to erlotinib in vivo, reminiscent of p.E322K we reported previously^6^, providing direct evidences for *MAPK1* mutant-driven erlotinib sensitivity by both *MAPK1*p.D321N and p.E322K in HNSCC. Our findings from this functional drug annotation study, together with our previous erlotinib exceptional response associated with *MAPK1*p.E322K provide multiple levels of evidences, supporting *MAPK1*-based precision clinical trials using erlotinib in HNSCC settings. Based on the TCGA-HNSCC cohort (*N* = 512)^9,10^, and the Johns-Hopkins HNSCC cohort (*N* = 32)^15^, the mutation rates of these two erlotinib-sensitive mutations range from 1.6 to 3.1% in HNSCC, which may account up to ~10,980–21,975 HNSCC patients per year (based on 0.7 million new cases of HNSCC per year in 2018 (refs ^9,10,15^)). Our current finding on erlotinib sensitivity of *MAPK1*p.D321N, together with the *MAPK1*p.E322K mutation in an erlotinib exceptional responder provide evidences for further investigations of these two mutations in clinical settings in HNSCC.

## Discussion

Despite extensive genomic characterization of HNSCC, precision medicine indications for this aggressive cancer remain limited. The only precision medicine choice for HNSCC follows that of FDA pan-cancer approval of larotrectinib for any solid tumors with *NTRK* fusions, which is anticipated to be most relevant for salivary gland tumors among all HNSCC (ref. ^16^). As of today, though EGFR-targeted therapy has been approved for HNSCC since 2006, the actual “precision way” of using EGFR inhibitors for HNSCC remains poorly defined.

We have previously reported findings with the first exceptional responder of HNSCC for EGFR inhibitor, whose tumor harbored *MAPK1*p.E322K mutation, which was then subsequently proven to confer heightened sensitivity to erlotinib in vivo^6,7^. Here, we sequenced 105 HNSCC tumors from Hong Kong, and we identified two other *MAPK1* mutations: p.R135K and p.D321N, in recurrent HNSCC patients (both have AJCC stage T4a diseases with disease recurrences). More importantly, functional analyses demonstrated that both mutations upregulated p-EGFR (Y1173) in vitro and in vivo, as compared to *MAPK1*-WT. Specifically, *MAPK1*p.D321N mutation, which caused a high level of EGFR activation in HNSCC cells, conferred significant sensitivity to erlotinib in vivo, concordant with our exceptional responder report for *MAPK1*p.E322K mutation^6,7^. Such demonstrated functional similarities between *MAPK1*p.D321N mutation and *MAPK1*p.E322K (in the erlotinib exceptional responder^6,7^), in terms of driver activities, EGFR hyperactivating capabilities, and erlotinib sensitivities, do provide direct evidence for potential use of the EGFR inhibitor, erlotinib, for *MAPK1*p.D321N and p.E322K-mutated HNSCC in a precision manner. Further clinical trial is warranted for translating these findings to clinical utility. Lastly, it is worth investigating if tumors of other cancer types bearing *MAPK1*p.D321N and *MAPK1*p.E322K mutations are also erlotinib sensitizing or not. These may include cervical cancer, bladder cancer, and lung SCC based on our pan-cancer *MAPK1* mutation mapping (Fig. 1b). Estimation for HNSCC alone, ~10,980–21,975 HNSCC patients/year may bear these two erlotinib-sensitive mutations with potential therapeutic benefits (based on a 1.6–3.1% mutation rate and 0.7 million new HNSCC cases per year in 2018 (refs ^9,10,15^)).

## Methods

### Tumor samples and targeted sequencing

*MAPK1*-targeted sequencing was analyzed using the IonS5 platform and Ion Reporter (ThermoFisher Scientific, USA). Clinical ethics approvals were obtained from the Research Ethics Committee of the Hospital Authority (University of Hong Kong/Hong Kong East Cluster; Joint Chinese University of Hong Kong–New Territories East Cluster; Kowloon West Cluster), Hong Kong SAR.

### Retroviral vectors and Infection

pMXs-puro-*MAPK1*-WT, pMXs-puro-*MAPK1*p.D321N, and pMXs-puro-*MAPK1*p.R135K were generated by site-directed mutagenesis with Sanger sequencing confirmation. Vectors were transfected into Plat-A retroviral production cells (Cell Biolabs, USA) for 3 days, and retroviruses were collected and used for infection for FaDu cells (purchased from ATCC, USA) as previously published^6^. Expression of *MAPK1* mutants were confirmed by western blotting. Infected cells were plated at 1.2 × 10^5^ cells/well in 48-well plate, and subjected to 5% FBS growth conditions for 96 h. MTT assays were then performed to determine the driver activity for growth vs. EGFP control. Cumulative results from three independent experiments with a total *N* ≥ 14 wells were plotted.

### Western blotting

Cell lysates were collected and analyzed by 8% SDS–PAGE, followed by primary antibody and secondary antibody incubations, and subsequent chemiluminescence development as previously^17^. Antibodies for p-EGFR (Y1173) is from Abcam UK (cat. ab32578, 1:1000), total EGFR (cat. 4267, 1:1000), p-MAPK(T202/Y204) (cat. 9101, 1:2000), and total MAPK (cat. 9102, 1:2000) were purchased from Cell Signaling Technologies, USA. Β-Actin antibody (cat. sc-69879, 1:3000) was purchased from Santa Cruz, USA. GOAT X RABBIT-HRP (Bio-rad, cat. 170-6515, 1:2000) or GOAT X MOUSE-HRP (Bio-rad, cat. 170-6516, 1:2000) were used for secondary antibody incubation depending on the source of primary antibodies. All blots were derived from the same experiment and were processed in parallel.

### Immunohistochemistry

Immunohistochemistry (IHC) was performed as previously described^18^. The VECTASTAIN Elite ABC Universal PLUS Kit Peroxidase (Horse Anti-Mouse/Rabbit IgG) (cat. PKK-8200) was used for IHC. Cytokeratin mouse antibody (DAKO, cat. M3515, 1:500) and Anti-EGFR (phosphor Y1173) antibody [E124] (Abcam, cat. ab32578, 1:100) were used as primary antibodies.

### In vivo experiments

All animal experiments were approved by the University Animal Experimentation Ethics Committee of the Chinese University of Hong Kong. Isogenic FaDu cells were infected with *MAPK1*-WT and *MAPK1* mutants by retrovirus, and were injected into nude mice subcutaneously (8 × 10^5^ cell per mouse, age of 4–5 weeks). Mice injected with respective infected FaDu cells were randomized into erlotinib treatment group or vehicle group (two tumors born by each mice, four mice per group). Treatment started at day 6 after injection with six doses per week until day 15. Erlotinib (dissolved in 10% HP-β-CD) or vehicle control were administrated orally at a dose of 50 mg/kg. Tumor volume was monitored and calculated by the equation: length × width^2^/2 repeatedly for five consecutive days as indicated by the arrows on the *X*-axis of Fig. 2a. The mice were sacrificed at the end point of the experiment.

### Statistical analysis

Student *t*-test (with non-parametric Mann–Whitney test, two-sided) were performed using the GraphPad Prism software.

### Reporting summary

Further information on research design is available in the Nature Research Reporting Summary linked to this article.

## Supplementary information

Supplementary Information

Reporting Summary

## Data Availability

The data/reanalysis that support the findings of this study are publicly available online at https://www.cbioportal.org/, https://cancer.sanger.ac.uk/cosmic and https://www.ncbi.nlm.nih.gov/Structure/index.shtml. All other data supporting the findings of this study are available from the corresponding author on request.
