# Supplementary material for: Erlotinib sensitivity of MAPK1p.D321N mutation in head and neck squamous cell carcinoma
Source: NPJ Genom Med. 2020 Apr 20;5:17. doi: 10.1038/s41525-020-0124-5 (PMC7171136; doi:10.1038/s41525-020-0124-5)
Supplement: Supplementary file 1 — Supplementary Information [file 41525_2020_124_MOESM1_ESM.pdf]

Supplementary Figure 1

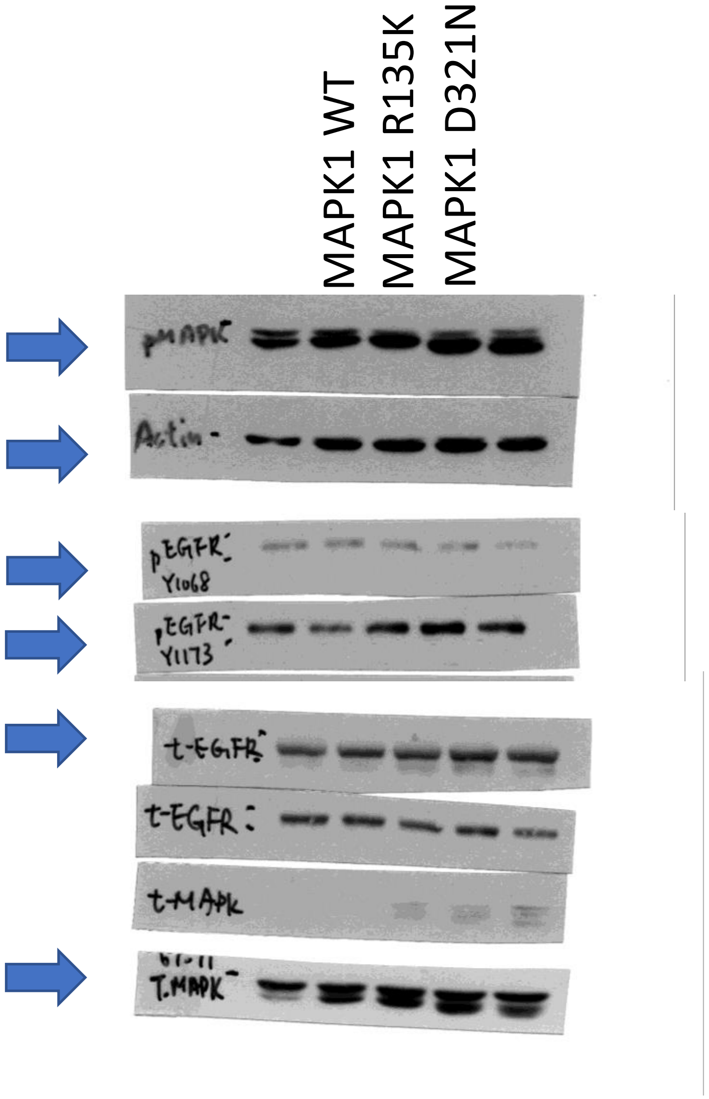

Supplementary Figure 1 - The un-cropped image of the western blotting film shown in Fig. 1(g). Arrows indicate the bands used.
